# Evaluation of the Antiangiogenic Activity of Thymus serpyllum Aerial Parts Extracts Using the Chorioallantoic Membrane Assay

**DOI:** 10.7759/cureus.99591

**Published:** 2025-12-18

**Authors:** Fouzia Latif, Kong Qun, Mingjing Lu

**Affiliations:** 1 School of Medicine, The Faculty of Medicine, Qilu Institute of Technology, Jinan, CHN

**Keywords:** angiogenesis, blood vessels, chorioallantoic membrane assay, fertilized chicken eggs, thymus serpyllum

## Abstract

Background and aim: Unregulated excessive angiogenesis is a hallmark of many diseases, including cancers, rheumatoid arthritis, and diabetic retinopathy. Plant *Thymus serpyllum* is well-known for its wide range of pharmacological effects. This study aimed to determine the in vivo antiangiogenic activity of *Thymus serpyllum* in fertilized chicken eggs using the chorioallantoic membrane (CAM) assay.

Materials and methods: The plant *Thymus serpyllum* aerial parts extract was prepared by maceration. The fourth-day fertilized chicken eggs were incubated. On the fifth day, eggs were windowed, and albumin was withdrawn. On the sixth day, 0.5% and 1% extracts were applied to the chorioallantoic membrane area, and the eggs were then reincubated. On the seventh day, CAM vasculature was imaged, and the diameter (primary, secondary, and tertiary), height (Abbott curve), and orientation pattern with amplitude of (angular spectrum) of blood vessels were measured by MountainsSPIP software (Besançon, France: Digital Surf). A relative comparison of the control and extract treatment groups was conducted.

Results: The control group exhibited a well-developed vascular network, characterized by dense and prominent branching of blood vessels with increased diameter; a steep Abbott curve, indicating increased complex vasculature and surface roughness; and a broad angular spectrum, revealing high amplitude and uniform orientation of blood vessels. In contrast, the 0.5% and 1% extracts reduced the dense vasculature, complexity, diameter of primary, secondary and tertiary blood vessels, uniform directionality, and amplitude of blood vessels in the angular spectrum. The 1% extract showed fading of tertiary blood vessels. Both extracts showed a gradual decline in the Abbott curve, but 1% extract showed a decrease in the height of blood vessels.

Conclusion: *Thymus serpyllum* 0.5% and 1% extracts exerted antiangiogenic effect on blood vessels in fertilized chicken eggs by reducing the vascular network, surface roughness, amplitude, and orientation pattern of blood vessels with a decrease in height by 1% extract. The antiangiogenic activity of *Thymus serpyllum* can be utilized therapeutically to treat various diseases associated with unregulated neovascularization.

## Introduction

Angiogenesis is the process by which new blood vessels are formed from pre-existing blood vessels, and it is clinically critical, as it can be either physiological or pathological [1]. Excessive and uncontrolled angiogenesis leads to various diseases, while decreased and insufficient angiogenesis below the normal level results in impairment of normal physiological conditions, such as tissue damage [2]. Angiogenesis at the physiological level is required for the healing of wounds, ischemic responses, reproduction, and development [3]. In pathological angiogenesis, uncontrolled neovascularization leads to different diseases, such as diabetic retinopathy, arthritis, cancer, and psoriasis. The growth and metastasis of cancer are caused by angiogenesis [4]. In both acute and chronic heart failure, neovascularization leads to vascular damage due to the presence of circulating endothelial cells in blood vessels [5]. In arthritis, the uncontrolled angiogenesis initiates an inflammatory response in the synovial fluid [6]. During angiogenesis, inflammatory cells migrate and positively regulate neovascularization, thereby supporting the angiogenic process [7]. In physiological angiogenesis, the shape of blood vessels is well-defined, and blood vessels grow properly to fulfill the metabolic needs of the body, while in pathological angiogenesis, the blood vessels are irregularly shaped [8]. Several different proteins with pro-angiogenic activities regulate the process of angiogenesis. Pro-angiogenic proteins include fibroblast growth factor (FGF), vascular endothelial growth factor (VEGF), platelet-derived growth factor (PDGF), transforming growth factors, insulin-like growth factor, angiopoietins, and various chemokines. Among proangiogenic proteins, VEGF is the most vital regulator of the angiogenesis process [9]. Physiological and pathological angiogenesis originate through the same phenomena, but during the pathological process, the proangiogenic factors are in excess [10]. Plant-derived products are safer for treating angiogenesis-associated diseases than synthetic drugs [11]. Natural products have proven their efficacy by exerting their antiangiogenic effects in various diseases [12]. Plant *Thymus serpyllum* is a perennial shrub, belonging to the Lamiaceae family, and is commonly known as wild thyme. This plant's raw material, essential oil, is rich in biologically important polyphenolic compounds that can be utilized in many pharmaceutical, food, and cosmetic products. Although the chemical composition of *Thymus serpyllum* varies according to collection region, its essential oil mainly contains carvacrol, thymol, pinene, terpinyl acetate, linalool, geraniol, borneol, caryophyllene, isobutyl acetate, 1,8-cineole, and citronellal [13]. This plant is famous for its medicinal effects, including antioxidant, anti-inflammatory, antimicrobial, and anticancer. Traditionally, *Thymus serpyllum* is used as a home remedy to treat flu, headache, cold, indigestion, ulcer, kidney diseases, diabetes, respiratory diseases, and gastrointestinal diseases [14]. Thymus essential oil is famous worldwide due to its aromatic and medicinal uses in mouthwashes and gargles for disinfection. *Thymus serpyllum* is a famous herbal tea. *Thymus serpyllum* possesses nutritional properties and reflects consumer needs by being used in different sauces, seasonings, and vitamin supplements [15]. This study explores the antiangiogenic activity of *Thymus serpyllum* in fertilized chicken eggs. The current study aimed to evaluate, in vivo, the antiangiogenic activity of *Thymus serpyllum *extract in fertilized chicken embryos using the chorioallantoic membrane (CAM) assay, which involves observation of the blood vessel network; branching of blood vessels with their distribution; and determination of the diameter, height, surface roughness, orientation pattern, and amplitude of blood vessels. This study seeks to compare the relative effects by taking one representative image of plant extract treatment groups with the control group in fertilized chicken eggs using the CAM assay. 

## Materials and methods

Preparation of extracts

The plant aerial (stem, flower buds, and flowers) parts were taken from a herbal company, Sichuan Wekeqi Biotechnology (Chengdu, China). The plant parts were verified by a botanist, Professor Nan Xu, at Qilu Institute of Technology (Jinan, China). The voucher (3857) was deposited in the medical department herbarium. Plant parts were air-dried and crushed to make powder, then 100 g of powder was dissolved in methanol solvent 1 L for 72 h to make the methanolic extract by the maceration process. The whole material was filtered after 72 h, and evaporation of the filtrate was carried out by rotary evaporator at less than 40°C under reduced pressure. Then the liquid extract was dried in air, and the dark brown extract was stored at 4°C in a refrigerator for subsequent experimental utilization.

Sterilization of the experimental area

The laboratory area was sterilized prior to the experiment using the fumigation method. First, 4 g KMnO_4_ was added to the sand vessel, then 8 mL formalin was added, and the sand vessel was kept in the laboratory area for 20 min.

Chicken egg incubation

Fertilized chicken eggs on the fourth day of incubation were obtained from a commercial hatchery (Jinan, China). The outer surface contamination of chicken eggs was removed by spraying with 70% ethanol and gently rubbing the outer surface with cotton. The excess use of spray was avoided to prevent the diffusion of 70% ethanol from the pores of chicken eggs into embryos, so that the pure effects of treated CAMs with tested plant drugs could be observed. Later, the chicken eggs were incubated at 37°C, 60-70% humidity.

CAM assay

In the laminar flow hood, the chicken embryos were divided into a control group and two treatment groups, with plant extracts at 0.5% and 1%, consisting of eight eggs in each group, on the fifth day of incubation. In the eggshells, windows of 2 cm in diameter were made by rupturing the eggshell and its underlying membrane at the broader outer end of the chicken egg under aseptic conditions. The albumin (approximately 4-5 mL) was aspirated using a 21-gauge disposable syringe cannula to facilitate easy assessment of the chicken embryo and improve its manipulation. Later, the window was sealed with sterile parafilm. The same procedure was repeated with all chicken eggs, and then the eggs were incubated at 37°C and 60-70% humidity. Chicken eggs were kept in the incubator with upright windows for 24 h on the fifth day of incubation. Then, windows of chicken eggs were opened on the sixth day and 150 µL of plant *Thymus serpyllum* methanolic extract at 0.5% and 1% concentrations was administered in the CAM area by disposable syringe, and the window was resealed, and the chicken eggs were again incubated on the sixth day. After 24 h, the parafilm covering the windows of chicken eggs was removed to open the windows, and blood vasculature growth in developing CAMs was observed. Images of the control and two treatment groups (0.5% and 1% extracts) were captured with a digital camera, and one representative image from the control and from each of the two treatment groups was imported separately into MountainsSPIP software (Besançon, France: Digital Surf) for 3D quantification of blood vessels. This allowed measurement of the diameters of primary, secondary, and tertiary blood vessels; measurement of blood vessel height and surface roughness using the Abbott curve; and measurement of blood vessel orientation pattern and amplitude using the angular spectrum [16].

Statistical analysis

The relative descriptive comparison among different groups was made without replicates by taking one representative image of each group (n=1); therefore, no statistical analysis with a p-value was required. One representative image, each of the control group and two treatment groups, including the 0.5% extract group and the 1% extract group, was selected and imported into MountainsSPIP software (Besançon, France: Digital Surf) for 3D quantification of blood vessel diameter, Abbott curve, and angular spectrum. The MountainsSPIP showed the relative comparative measurements of diameter, height, and amplitude in arbitrary units or pixel intensity gradients rather than absolute measurements.

## Results

**Figure 1:**
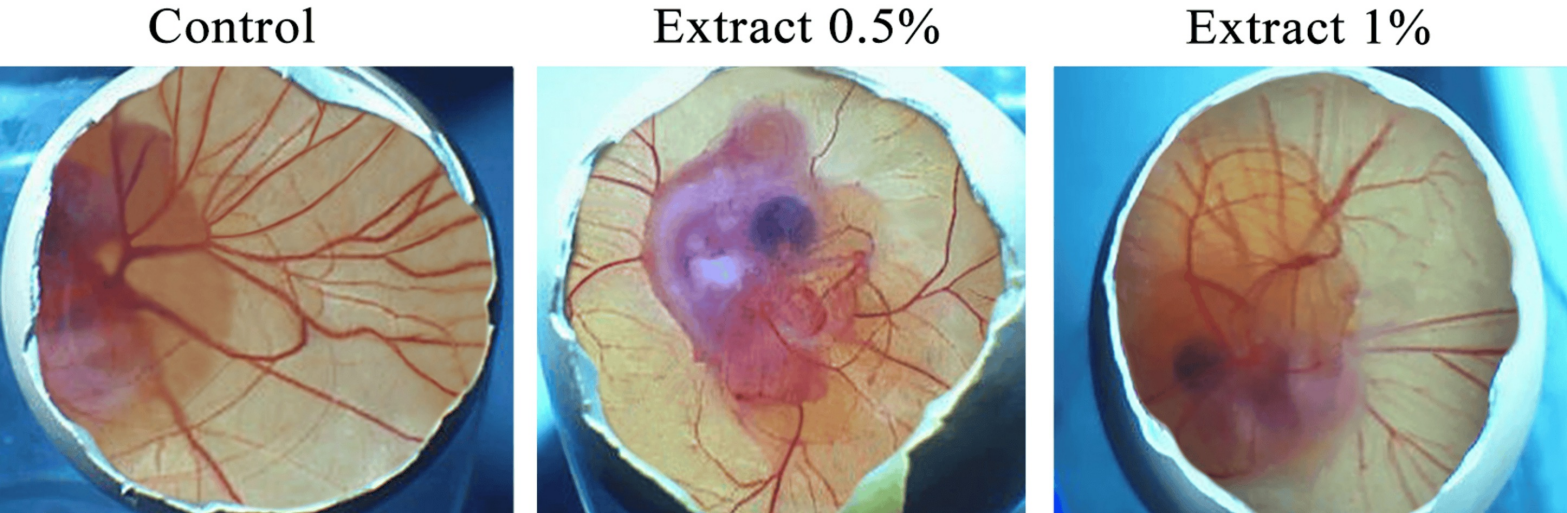
Thymus serpyllum extracts effect on vascular network and branches of blood vessels in chorioallantoic membrane area of fertilized chicken eggs. In the fertilized chicken eggs treatment groups of 0.5% and 1% extracts, the vascular network, its distribution, and branches of blood vessels dividing into primary, secondary, and tertiary blood vessels were reduced compared with the control group. One representative image of each experimental group of fertilized chicken eggs was taken.

The control group in chicken embryo showed a well-developed vascular network and a dense CAM area, with the main trunk dividing into primary, secondary, and tertiary blood vessels that were distributed throughout the egg yolk. The blood vessels were multidirectionally spread. Whereas *Thymus serpyllum* extracts at concentrations of 0.5% and 1% in treated CAMs exhibited a significant antiangiogenic effect by reducing CAM area, trunk formation, and blood vessel architecture. The blood vasculature was not widely spread, and a reduced multidirectional blood vessel network was seen in the egg yolk area. *Thymus serpyllum* extract at 0.5% decreased the number of prominent visible primary, secondary, and tertiary blood vessels in the treated chicken embryo CAM compared with the control. In contrast, those treated with 1% extract showed the most effective decrease in the blood vessel network. The primary and secondary blood vessels were prominent, but the tertiary blood vessels were fading (Figure 1).

**Figure 2:**
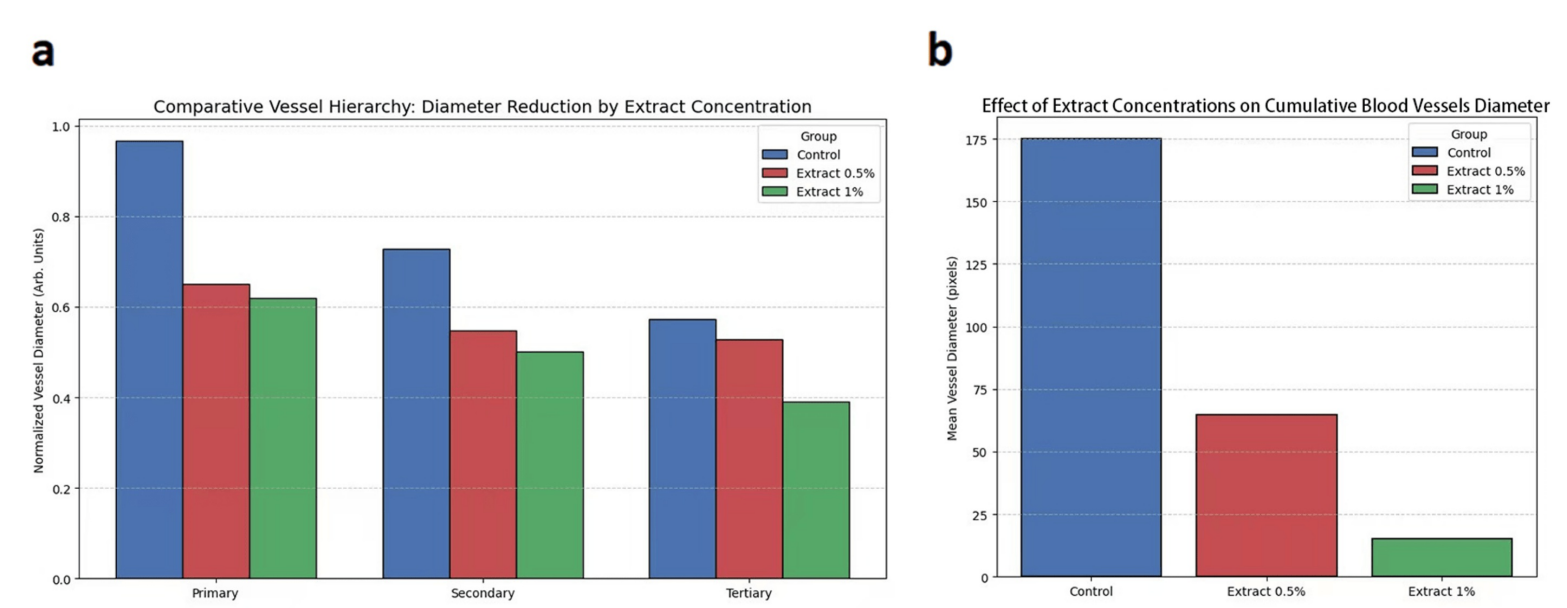
Effect of Thymus serpyllum extract on the diameter of blood vessels in fertilized chicken eggs. Fertilized chicken egg groups treated with 0.5% and 1% *Thymus serpyllum *extracts showed (a) a decrease in the diameter of primary, secondary, and tertiary blood vessels and (b) a decrease in cumulative blood vessel diameter (sum of primary, secondary, and tertiary vessels) using the chorioallantoic membrane assay (n=1; therefore, no statistical analysis with p-values was applied, as only one representative image was taken for each experimental group).

In the control group, the diameter of primary, secondary, and tertiary blood vessels was significantly higher, which indicated a remarkable angiogenic activity in the CAM area of chicken embryos. Whereas the 0.5% and 1% extracts treated CAM groups showed a decrease in the diameter of primary, secondary, and tertiary blood vessels. The cumulative diameter, which means the sum of primary, secondary, and tertiary blood vessels, was also decreased in 0.5% and 1% extracts treated group than the control group. The most significant reduction in blood vessel diameter was observed with the 1% extract (Figures 2a, 2b).

**Figure 3:**
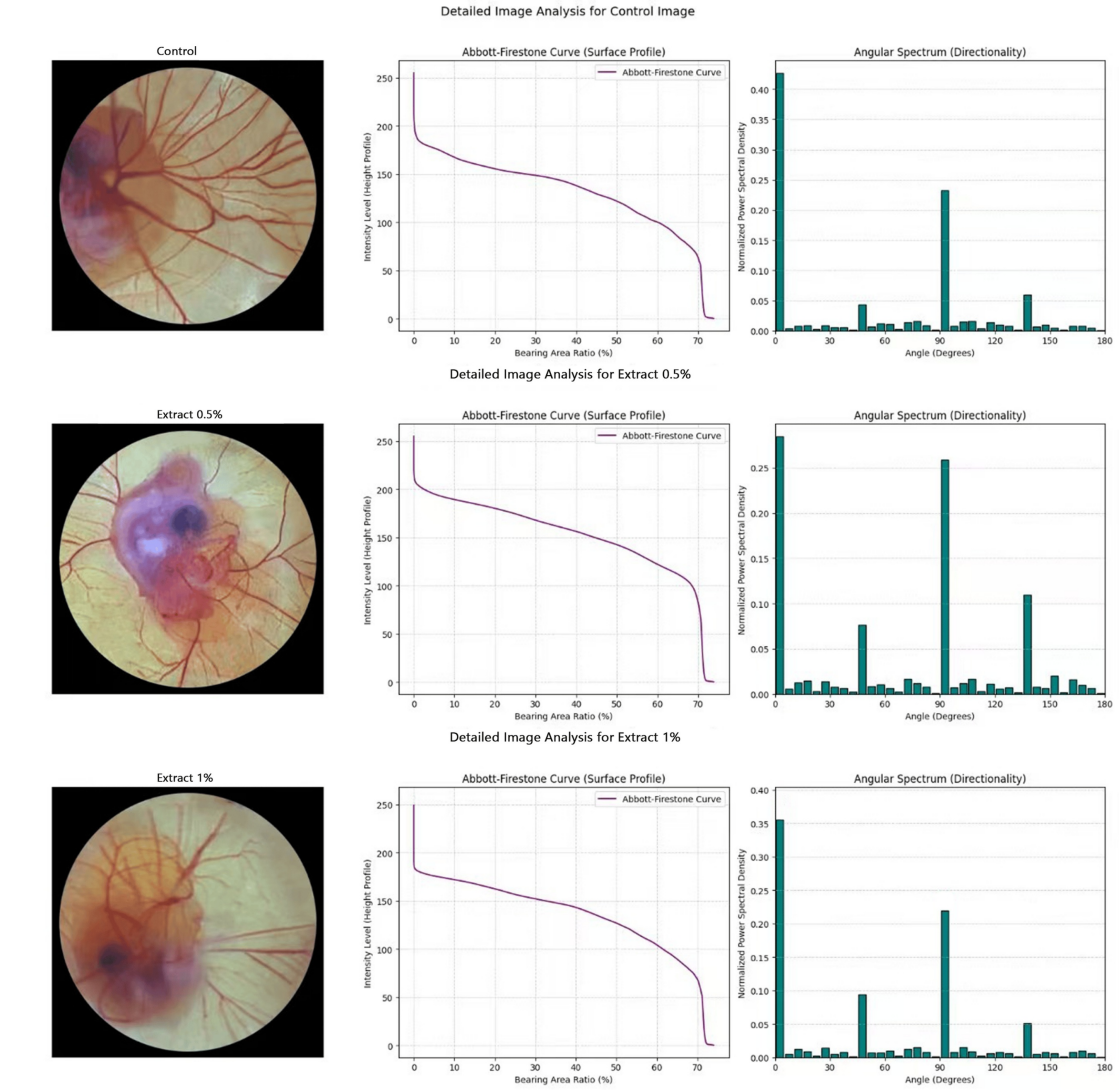
Effect of Thymus serpyllum extracts on blood vessel diameter, height, and surface roughness (Abbott curve) as well as on amplitude and orientation pattern (angular spectrum) in fertilized chicken eggs using the CAM assay. (a) The control group showed a steeper decline of the Abbott curve, revealing an increase in height and surface roughness of blood vessels; the angular spectrum revealed high amplitude of blood vessels with a broad angle of orientation pattern. (b) The 0.5% extract group showed a gradual decline of the Abbott curve, a decrease in surface roughness without affecting the height of blood vessels, and a narrow angular spectrum with decreased amplitude and altered orientation pattern of blood vessels. (c) The 1% extract group showed a gradual decline of the Abbott curve, decreases in both height and surface roughness of blood vessels, and a narrow angular spectrum with reduced amplitude and altered orientation pattern of blood vessels (n=1; therefore, no statistical analysis with p-values was applied, as only one representative image was used for each experimental group). CAM: chorioallantoic membrane

In the control group, the Abbott curve starts with a higher height value, above the 250 value on the Y-axis. The Abbott curve showed a steeper decline, indicating the dense complex blood vessels, which had more surface roughness. The Abbott curve for treated CAMs with *Thymus serpyllum* extracts at 0.5% and 1% concentrations showed a gradual curve, indicating the formation of more flattened blood vessels with reduced surface roughness. The 0.5% extract showed no effect on reducing the height of blood vessels, whereas the 1% extract decreased the height of blood vessels starting at 250 value on the Y-axis, compared to the control group. The angular spectrum of the control group revealed blood vessel orientation with multiple peaks at broad angles, ranging from 0° to 180°, indicating a complex blood vascular network with numerous branches. The control group showed a high value of normalized power spectral density at 0° (about 0.45), indicating a uniform, well-oriented vascular architecture with a high amplitude of the blood vessels. The angular spectrum in the treatment groups of *Thymus serpyllum* 0.5% and 1% extracts appeared narrow due to less complex branching of blood vessels. The 0.5% extract at 0° exhibited a relatively higher decrease in normalized power spectral density, about 0.30, and some slightly high values from 30° to 170°, which reveals the disorganized blood vessels orientation. The 1% extract showed a value of normalized power spectral density, slightly above 0.35 at 0°, with a slightly higher value at around 50°, compared to the control group. The angular spectrum of 1% showed a narrow angular spectrum with less amplitude due to other reduced values of normalized power spectral density, which revealed the strong antiangiogenic effect of 1% extract by showing regression of blood vessels with consistent loss of blood vessels orientation (Figure 3).

## Discussion

The medicines derived from natural sources have been used for hundreds of years. Plants are being used as traditional medicines in both the East and the West, across underdeveloped and developed countries [17]. Instead of the routine use of drugs from the synthetic source, the plant medicines are still considered famous because of easy access, few adverse reactions, and cost-effectiveness of plant drugs [18]. Natural plant extracts exhibit effective antiangiogenic activity, helping to prevent various diseases, including cancers, that are caused by neovascularization [19]. Plant *Thymus serpyllum* is well-known for its multiple pharmacological activities, nutritional value, and its use in the cosmetic and pharmaceutical industry. Although *Thymus serpyllum* has demonstrated its efficacy as an antioxidant, antibacterial, anti-inflammatory, and anticancer agent, no study has investigated its antiangiogenic effect to date. The current study aimed to explore the in vivo antiangiogenic effect of *Thymus serpyllum* extracts, 0.5% and 1% from the aerial parts, in fertilized chicken embryos using the CAM assay by observing the vascular network and branches of blood vessels and their distribution area, and by measuring the diameter, height, surface roughness, amplitude, and orientation pattern of blood vessels. There are many in vivo assays, but the CAM assay was employed because the chicken embryo's CAM has a dense capillary network, facilitating easy visualization of the biomaterial's effects on blood vessels. The CAM assay is a simple, cost-effective procedure that enables the blood vessel 3D quantification by MountainsSPIP software [20]. The fertilized chicken eggs, due to their characteristic chorioallantoic membrane, have sparked the interest of researchers in bioengineering and biomedicine, leading them to study the effects of drugs on blood vessel formation to assess their angiogenic activity [21]. In the developing avian embryo, the chorioallantoic membrane, attached to the eggshell, is highly vascularized and performs multiple functions during embryonic growth and development. It is essential for the exchange of nutrients and also functions as a breathing organ to exchange gases through the eggshell pores between the embryo and the external environment. The chorioallantoic membrane serves as the excretory organ for removing minerals and as a reservoir for waste products [22]. When the CAM grows, it forms a well-developed, multibranched, and dense blood vascular network that facilitates the growth and development of the embryo [23]. An image-probing system, the scanning probe image processor (SPIP) - referred to as MountainsSPIP - was used to determine the 3D quantification of blood vessels in the control group and *Thymus serpyllum *extract-treated CAM groups of fertilized chicken eggs. MountainsSPIP software quantifies 3D angiogenesis from CAM images by using a specified algorithm, measurement calibration, and commands to determine the diameter, height, surface roughness, orientation pattern, and amplitude of blood vessels [24]. From the CAM images, MountainsSPIP generates the Abbott curve, which provides a graphical representation of blood vessel surface roughness on the CAM, indicating variations in vessel height. The Abbott curve quantifies minor changes in the blood vessels of the CAM surface during blood vessel development, resulting from treatment [25]. The angular spectrum using MountainsSPIP software indicates the orientation pattern of the blood vessels and measures the amplitude of the blood vessels to reveal the density and directionality of the blood vessels [26]. In the current study, the blood vessel network in the control group was well-distributed throughout the egg yolk area of fertilized chicken eggs. The blood vessels were multiple-branched and highly complex, with prominent primary, secondary, and tertiary blood vessels, which is a characteristic requirement of a natural blood vessel network. *Thymus serpyllum* extracts at 0.5% and 1% demonstrated antiangiogenic activity by reducing the multibranched blood vessel network and decreasing the number of primary, secondary, and tertiary blood vessels compared to the control group. The most pronounced antiangiogenic effect with 1% extract was seen, which showed fading of the tertiary blood vessel (Figure 1). In the control group, the increase in diameter of primary, secondary, and tertiary blood vessels was seen, which indicated the well-developed and mature dense blood vessels resulting from angiogenic activity. While in the treated CAM groups of 0.5% and 1% extracts, a decrease in the diameter of primary, secondary, and tertiary blood vessels was observed compared to the control group, which is attributed to its antiangiogenic activity due to the presence of less dense and immature blood vessels in the extract-treated CAM groups. The cumulative diameter of blood vessels (primary, secondary, and tertiary vessels) was also reduced in the 0.5% and 1% extract treatment groups compared to the control group. But the extract 1% remarkably decreased the diameter of blood vessels compared to the extract 0.5% (Figures 2a, 2b). In the control group, the Abbott curve is steeper, indicating a widespread and uniform distribution of dense blood vessels with a remarkable height and increased surface roughness, occupying a large area of the egg yolk. While the *Thymus serpyllum* extracts, 0.5% and 1% treated CAMs groups showed a gradual decrease of the Abbott curve, revealing a decrease in surface roughness of blood vessels with low density, which is attributed to the antiangiogenic activity of the extracts. However, the 1% extract remarkably decreased the height of the Abbott curve, revealing the low height of blood vessels; whereas, the extract 0.5% did not reduce the height of the Abbott curve (Figure 3). In the control group, the angular spectrum showed a high amplitude and displayed a preferred orientation of blood vessels at an angle of 0°. In comparison, the 0.5% extract treatment group showed the partial loss of uniformity in blood vessel directionality and prominently reduced amplitude at 0°. The angular spectrum of extract 1% also showed a consistent disorganized orientation pattern of blood vessels with less amplitude at 0°. Although the angular spectrum of both 0.5% and 1% extracts was observed to be narrower compared to the control, but the 1% extract showed a stronger regression of blood vessel directionality (Figure 3). It might be possible that blood vessels were unevenly collapsed, and there was a disturbance in the pattern of blood vessels. This directionality can be reduced when a higher dose can damage the cell layer of blood vessels. Some collapse of blood vessels was observed in the CAM area near the chicken embryo without a toxic effect on the embryo (Figure 1). The current study employed two different concentrations of *Thymus serpyllum* extract to evaluate whether the higher dose causes toxicity, while also observing the dose-dependent antiangiogenic effect. The current study results confirmed that *Thymus serpyllum* extracts at 0.5% and 1% concentrations demonstrated antiangiogenic activity by reducing the blood vessel network and the diameter of primary, secondary, and tertiary blood vessels. Both plant extracts showed a gradual decrease in the Abbott curve slope and decreased surface roughness of blood vessels, while the 1% extract decreased the height of blood vessels by decreasing the height of the Abbott curve. Both extracts showed an irregular orientation pattern with reduced amplitude of blood vessels in the angular spectrum. Although the 0.5% extract effectively reduced the amplitude of blood vessels in the angular spectrum, 1% extract showed the most pronounced reduction in the blood vascular network, its distribution area, diameter, height, and surface roughness of blood vessels. Extract 1% also showed more loss of blood vessels' uniformity in orientation pattern. On the basis of these results, extract 1% showed the most remarkable antiangiogenic effect compared to the 0.5% extract, showing pronounced regression of blood vessels.

Limitations of the study

Although *Thymus serpyllum* has not been previously reported for in vivo toxicity, the current study is the first to evaluate its antiangiogenic activity in a developing chicken embryo model. However, no preliminary experiment was conducted to assess the safety and potential toxicity of *Thymus serpyllum* in developing embryos in chicken eggs. The current study investigated the in vivo antiangiogenic activity of *Thymus serpyllum *extract; however, this study lacks the in vitro evaluation of *Thymus serpyllum* extract so that a comparison can be made. In vitro, the detailed mechanism of antiangiogenic activity should be studied by employing angiogenic and antiangiogenic markers. Angiogenesis at the pathological level drives cancer progression; therefore, cell lines from different cancer types should be used to evaluate which cancer cells the *Thymus serpyllum* extract exerts a significant antiangiogenic effect on. Another limitation of this study is the use of a large number of fertilized chicken eggs to achieve the best image of CAM, which displays the blood vessel area to observe the complexity of blood vessels and facilitates the measurement of diameter, amplitude, and height of blood vessels.

## Conclusions

Our study findings revealed that plant *Thymus serpyllum* aerial parts extracts, at concentrations of 0.5% and 1%, showed antiangiogenic activity in the CAM area of fertilized chicken embryo by reducing the blood vessel network with distribution area, thereby decreasing the diameter of primary, secondary, and tertiary blood vessels. In the plant extracts-treated CAM groups, there was a gradual decrease in the slope of the Abbott curve, due to less dense and reduced surface roughness of blood vessels. The amplitude and orientation pattern of blood vessels in the angular spectrum were also decreased. The height of blood vessels in the Abbott curve was reduced by 1% extract. Although the amplitude of blood vessels was prominently decreased by 0.5% extract. But the 1% extract most effectively decreased the vascular network, diameter, height, and surface roughness of blood vessels compared to the 0.5% extract. Therefore, the higher dose of extract at 1% concentration demonstrated the most remarkable antiangiogenic activity, and it was also non-toxic to the chicken embryo. The future in-depth study of the antiangiogenic mechanism of *Thymus serpyllum *should be employed to explore its therapeutic potential in various cancers and wound healing.

## References

[1] Kretschmer M, Rüdiger D, Zahler S (2021). Mechanical aspects of angiogenesis. Cancers (Basel).

[2] La Mendola D, Trincavelli ML, Martini C (2022). Angiogenesis in disease. Int J Mol Sci.

[3] Sedighi M, Namdari M, Mahmoudi P (2023). An overview of angiogenesis and chemical and physiological angiogenic factors: short review. J Chem Health Risks.

[4] Dudley AC, Griffioen AW (2023). Pathological angiogenesis: mechanisms and therapeutic strategies. Angiogenesis.

[5] Khurana R, Simons M, Martin JF, Zachary IC (2005). Role of angiogenesis in cardiovascular disease: a critical appraisal. Circulation.

[6] Marrelli A, Cipriani P, Liakouli V, Carubbi F, Perricone C, Perricone R, Giacomelli R (2011). Angiogenesis in rheumatoid arthritis: a disease specific process or a common response to chronic inflammation?. Autoimmun Rev.

[7] Jeong JH, Ojha U, Lee YM (2021). Pathological angiogenesis and inflammation in tissues. Arch Pharm Res.

[8] Emanueli C, Madeddu P (2006). Therapeutic angiogenesis: translating experimental concepts to medically relevant goals. Vascul Pharmacol.

[9] Omorphos NP, Gao C, Tan SS, Sangha MS (2021). Understanding angiogenesis and the role of angiogenic growth factors in the vascularisation of engineered tissues. Mol Biol Rep.

[10] Akbarian M, Bertassoni LE, Tayebi L (2022). Biological aspects in controlling angiogenesis: current progress. Cell Mol Life Sci.

[11] Fan TP, Yeh JC, Leung KW, Yue PY, Wong RN (2006). Angiogenesis: from plants to blood vessels. Trends Pharmacol Sci.

[12] Hoseinkhani Z, Norooznezhad F, Rastegari-Pouyani M, Mansouri K (2020). Medicinal plants extracts with antiangiogenic activity: where is the link?. Adv Pharm Bull.

[13] Jovanovic AA, Balanc B, Petrovic P, Pravilovic R, Djordjevic V (2021). Pharmacological potential of Thymus serpyllum L. (wild thyme) extracts and essential oil: a review. J Eng Process Manag.

[14] Salaria D, Rolta R, Lal UR, Dev K, Kumar V (2023). A comprehensive review on traditional applications, phytochemistry, pharmacology, and toxicology of Thymus serpyllum. Indian J Pharmacol.

[15] Jalil B, Pischel I, Feistel B (2024). Wild thyme (Thymus serpyllum L.): a review of the current evidence of nutritional and preventive health benefits. Front Nutr.

[16] Tabassum N, Aziz A, Ahmad B (2016). Evaluation of pharmacological effect of Teucrium stocksianum extract on angiogenesis using chorioallantoic membrane assay. Bangladesh J Pharmacol.

[17] Chaachouay N, Zidane L (2024). Plant-derived natural products: a source for drug discovery and development. Drugs Drug Candidates.

[18] Fokunang ET, Fonmboh DJ, Mballa RN (2020). Pharmacovigilance of natural herbal medicines research for efficacy, safety and quality assurance of phytomedicine products. J Complement Altern Med Res.

[19] Nunez CV, de Vasconcellos MC, Alaniz L (2022). Are natural products, used as antitumoral/antiangiogenic agents, less toxic than synthetic conventional chemotherapy?. Front Pharmacol.

[20] Ribatti D, Annese T, Tamma R (2020). The use of the chick embryo CAM assay in the study of angiogenic activiy of biomaterials. Microvasc Res.

[21] Chen L, Wang S, Feng Y (2021). Utilisation of chick embryo chorioallantoic membrane as a model platform for imaging-navigated biomedical research. Cells.

[22] Kundeková B, Máčajová M, Meta M, Čavarga I, Bilčík B (2021). Chorioallantoic membrane models of various avian species: differences and applications. Biology (Basel).

[23] Wan Z, Hirche C, Fricke F, Dragu A, Will PA (2025). Chick chorioallantoic membrane as an in vivo model for the study of angiogenesis and lymphangiogenesis. J Vasc Res.

[24] Omer I, Ur-Rehman M (2018). Investigation of diclofenac sodium on angiogenesis through blood assay. Medbiotech J.

[25] Rehman G, Sardar S, Alkhateeb MA (2022). Evaluation of functional and bioactive properties of crude gill extract of Tor putitora using different assays. Int J Food Sci Technol.

[26] Bashir Bashir, Farhan M, Qadir MI (2017). Effect of ginger extract on angiogenesis using CAM assay. Bangladesh J Pharmacol.

